# The Role of Sialic Acid-Binding Receptors (Siglecs) in the Immunomodulatory Effects of *Trypanosoma cruzi* Sialoglycoproteins on the Protective Immunity of the Host

**DOI:** 10.1155/2013/965856

**Published:** 2013-12-22

**Authors:** Alexandre Morrot

**Affiliations:** Institute of Microbiology, Federal University of Rio de Janeiro, CCS, Sala D1-035, Avenida Carlos Chagas Filho 373, Cidade Universitária, Ilha do Fundão, 21.941-902 Rio de Janeiro, RJ, Brazil

## Abstract

Chagas disease is caused by the protozoan parasite *Trypanosoma cruzi* and is an important endemic infection in Latin America. Lately, it has also become a health concern in the United States and Europe. Most of the immunomodulatory mechanisms associated with this parasitic infection have been attributed to mucin-like molecules on the *T. cruzi* surface. Mucins are high molecular weight glycoproteins that are involved in regulating diverse cellular activities in both normal and pathological conditions. In *Trypanosoma cruzi* infection, the parasite-derived mucins are the main acceptors of sialic acid and it has been suggested that they play a role in various host-parasite interactions during the course of Chagas disease. Recently, we have presented evidence that sialylation of the mucins is required for the inhibitory effects on CD4^+^ T cells. In what follows we propose that signaling via sialic acid-binding Ig-like lectin receptors for these highly sialylated structures on host cells contributes to the arrest of cell cycle progression in the G1 phase and may allow the parasite to modulate the immune system of the host.

## 1. *Trypanosoma cruzi* Infection and the Immunopathology of Chagas Disease

Chagas' disease or American trypanosomiasis is a tropical parasitic illness affecting nearly 20 million people in the Americas [1, 2]. The disease is caused by the protozoan flagellated parasite *Trypanosoma cruzi*, transmitted to humans by haematophagous insects known as triatomines (Reduviidae family). The complex life cycle of *T. cruzi* includes epimastigote and metacyclic trypomastigote stages in the insect vector and bloodstream trypomastigote and intracellular amastigotes in the vertebrate host [3]. In the latter, the *Trypanosoma cruzi* infects several cell types, including monocytes, fibroblasts, endothelial cells, and muscle cells [4–9]. This capacity to invade a wide range of host cells is associated with increased tissue inflammation and evokes a strong immunological response. This host protective response results from host tissue damage due to increased infiltration of leukocytes to the inflammatory sites, producing proinflammatory mediators, including cytokines, chemokines, and nitric oxide, among other factors [10–14]. Approximately 30% of infected patients develop symptoms of the disease in their lifetime; these include cardiomyopathy, neuropathies, and dilatation of the colon or esophagus [15].

The pathogenesis of Chagas disease is controversial and distinct hypotheses have been considered, including autoimmune manifestations and parasite-driven tissue damage [16–18]. Whatever is the case, it is accepted that the events occurring during the acute phase of *T. cruzi* infection determine the pathological features that arise later during the chronic phase of the disease [19]. The initial stages of the infection are characterized by the induction of nonspecific lymphoproliferation [20]. This phenomenon involves extensive polyclonal activation of lymphocytes. There is an increased frequency of immunoglobulin-secreting B cells with the typical isotype profile for IgG2a and IgG2b in peripheral lymphoid organs, and the majority of these polyclonal activated B cells secrete nonspecific antibodies with low affinity for *T. cruzi* antigens. The T cells are also polyclonally expanded in the course of infection, but it seems that the massive polyclonal activation targets the minor CD5B and *γδ* T lymphocyte subsets [21].

This polyclonal activation is believed to have a role in inducing autoimmune reactions during Chagas disease. There are numerous reports of *T. cruzi* antigens cross-reactive with heart and neural tissues [22–24], but these autoantibodies or autoreactive T cells are believed to play secondary roles in the pathogenesis of Chagas disease as the affinity of the peripheral lymphocyte repertoire with cross-reactive antigens is low due to the negative selection that they undergo during the process of central tolerance [25–27]. However, it seems that the polyclonal activation in Chagas disease has a role in the immunosuppressive mechanisms associated with *Trypanosoma cruzi* infection. As the activation and survival of lymphocytes are determined by competitive access to niches containing antigen and cytokines in lymphoid tissues, it is possible that the polyclonal activation of lymphocytes dampens the protective immune response by limiting the competitiveness of antigen-specific lymphocyte relative to the high frequency of polyclonally expanded T/B cells [28–30]. These events could account for the immunosuppression seen in both mice and humans in the acute phase of Chagas disease [8, 31–39]. In addition to these alterations in peripheral lymphoid organs, the thymus is also a target for parasite-induced changes of the host immune system. During the acute phase of the disease, severe thymic atrophy occurs, mainly due to apoptotic depletion of CD4^+^CD8^+^ double-positive (DP) thymocytes in the cortical area of the thymic lobules [40].

In spite of this depletion in the thymus, there is also an abnormal release of DP cells into the periphery, resulting in a more than 15-fold increase in DP cell numbers in subcutaneous lymph nodes. This premature thymic emigration of immature thymocytes is likely to be a result of alterations of the thymic microenvironment, with enhanced deposition of cell migration-related molecules such extracellular matrix (ECM) proteins and chemokines CXCL12 and CCL21, which can influence the migration of developing thymocytes during thymopoiesis [41–44]. Interestingly, we have shown that, in contrast to physiological conditions, the DP cells released into the periphery during the course of the infection acquire an activated phenotype similar to that described for activated single-positive T cells. Furthermore, we showed that the presence of activated DP cells in the periphery is correlated with the development of the severe clinical form of chronic human Chagas disease [40].

Despite the changes observed in the thymus during infection, we have shown that the intrathymic expression of the autoimmune regulator factor (Aire) and tissue-restricted antigen (TRA) genes is normal. In addition, expression of the proapoptotic Bim protein in thymocytes is unchanged, showing that the thymic atrophy has no effect on the checkpoints required for clonal T cell deletion. In a chicken egg ovalbumin- (OVA-) specific T cell receptor (TCR) transgenic system, the administration of OVA peptide to infected mice undergoing thymic atrophy promoted OVA-specific thymocyte apoptosis, further indicating that the negative selection process is normal during infection [40]. These findings indicate that the key intrathymic elements necessary for negative selection of thymocytes undergoing maturation during thymopoiesis remain functional during the acute chagasic thymic atrophy.

However, the fact that negative selection still operates in the thymus [40], which is also a locus of colonization by *T. cruzi* [45], may lead to newly generated T cells tolerant to the invading pathogen. During thymic colonization, the pathogen could be able to target the thymic DCs to promote clonal deletion of recycling activated pathogen-specific T cells that migrate from the thymus to clear the infection [46]. Although we showed that the key intrathymic elements responsible for negative selection of thymocytes are active during thymopoiesis [40], deletion of activated-recycling T cells specific for *T. cruzi *parasites in the thymus could play a role in the central tolerance mechanism promoting pathogen persistence.

## 2. The Immunosuppressive Effects of *Trypanosoma cruzi*-Derived Mucins

As in any chronic infectious disease, the *Trypanosoma cruzi* parasite has evolved the capacity to survive in its vertebrate host by weakening the host's immune response [35, 47–50]. In both humans and experimental models of *T. cruzi* infection, the acute phase of Chagas disease is marked by a state of immunosuppression [31–34, 36, 38]. This subversion of the host protective immune response at the beginning of infection in the acute phase of *T. cruzi* infection is responsible for the persistence of the parasite and the establishment of a chronic disease [51–53]. *T. cruzi *in fact provides a good example of such a strategy. T cells from infected mice show reduced IL-2 expression and low proliferative responses to mitogens [32, 33, 36]. In addition, CD4^+^ T cells from infected mice when activated by stimulation of the T cell receptor show enhanced apoptosis increasing the unresponsiveness of host immunity. These characteristics are indicative of an immunosuppressed state. This immunodeficiency is also characterized by reduced protective humoral responses [54–56].

**Figure 1:**
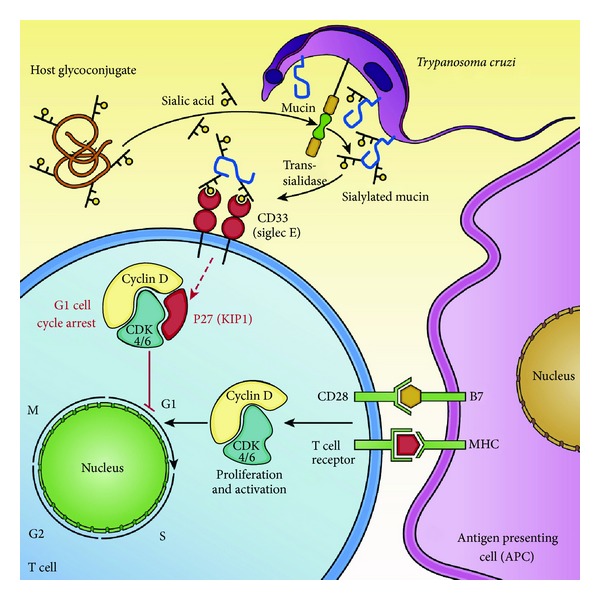
Model depicting the inhibitory effect of *Trypanosoma cruzi* sialoglycoproteins on T cell activation. Schematic diagram showing the sialylation of O-linked oligosaccharides of mucin-derived trypomastigotes mediated by the surface-associated *T. cruzi* parasite trans-sialidase. The parasite trans-sialidase, which can also be shed into the bloodstream or tissue interstitium after cleavage of its glycosylphosphatidylinositol (GPI) anchor by the action of a phosphatidylinositol-phospholipase C, transfers sialic-acid residues from host glycoconjugates to parasite mucins. It has been demonstrated in other studies that the *T. cruzi* parasite-derived mucins bind to mammalian host cell receptors such as the acid-binding Ig-like lectin receptor Siglec-E (CD33) and undermine host defence mechanisms. In CD4^+^ T cells, we showed that the Siglec-E receptor inhibits the mitogenic responses upon T cell receptor stimulation. The initiation of the G1 to S transition during antigenic/mitogenic T cell expansion is mediated by cyclin D and cyclin-dependent kinases CDK2 or CDK6, which are induced and together initiate the G1/S transition. We have shown that the G1/S transition is significantly inhibited by the sialyl terminal residues of *T. cruzi* mucins and we propose that this phenomenon is mediated by its interaction with the Siglec-E receptor. The interaction of CD4^+^ T cells with the sialylated form of the parasite mucin leads to induction of p27/Kip1, a member of the family of CDK inhibitors that negatively regulate the G1 to S transition, so damping T cell-mediated immune responses by inducing T cell cycle arrest.

The host-parasite interplay underlying the immunosuppression during Chagas disease has been elucidated. Independent studies have demonstrated that *T. cruzi* membrane glycoproteins are critical for damping host protective immunity. The parasite surface is covered by sialic acid residues which are transferred from host glycoconjugates to the terminal *β*-galactosyl residues of mucin-like molecules on its surface by a unique enzyme, the trans-sialidase [57–60] (Figure 1). These *T. cruzi *mucins are the most abundant glycoproteins on the surface of the parasite and consist of O-glycosylated Thr/Ser/Pro-rich proteins. The *T. cruzi* mucin-like molecules consist of a large repertoire of glycoproteins encoded by more than 800 genes comprising approximately 1% of the parasite genome [61–63]. These molecules play a key role in the invasion of the host and subversion of its immune system. It has been shown, for instance, that the sialylated mucins mask parasite antigenic determinants, thus protecting the parasite from host attack by anti-galactosyl antibodies and complement factor B [64–67].

The sialylated glycoconjugates have been shown to modulate the host dendritic cell function by suppressing the production of the proinflammatory cytokine IL-12, as well as T cell activation and proliferation in response to mitogens and antigens [68, 69] (Figure 1). These effects may involve action at the transcriptional level, since the *T. cruzi* mucins inhibit transcription of IL-2 gene [32, 33]. Moreover, other studies have shown that these sialoglycoproteins also inhibit early events in T cell activation such as tyrosine phosphorylation of the adapter protein SLP-76 and the tyrosine kinase ZAP-70 [36]. The inhibitory effects of the *T. cruzi* mucins were recently examined *in vivo*. We found that exposure of mice to exogenous *T. cruzi*-derived mucins during infection with *Trypanosoma cruzi* increased their susceptibility to infection and led to increased parasitemia and heart damage. These alterations were correlated with a lower frequency of IFN-*γ*-producing CD4^+^ and CD8^+^ T cell responses, in addition to decreased levels of both splenic IFN-*γ* and TNF-*α* cytokines [69]. These data indicated that the parasite mucins influence the course of the parasite-host interaction during the acquisition of cell-mediated adaptive immune responses, damping protective host responses in order to establish persistent chronic infections.

## 3. The Sialic Motifs of *Trypanosoma cruzi* Mucins Target Host Sialic Acid-Binding Receptors (Siglecs) during Parasite Immunomodulation

Sialic acids are found on all cell surfaces of a variety of organisms, including pathogens that interact with vertebrates [70]. In *Trypanosoma cruzi* infections, the sialylated glycoconjugates play important roles in the initiation, persistence, and pathogenesis of Chagas' disease [64–67, 71]. Their target receptors in the host have only been identified recently. There is evidence that sialylated Tc Muc can interact with Siglec-E (CD33) (Figure 1), a member of the Siglec family of sialic acid-binding Ig-like lectins found mainly on cells of the immune system [72, 73]. The Siglec receptors are structures similar to lectins and have variable specificity for sialic acid-containing ligands [74]. Siglecs have immunoreceptor tyrosine-based inhibitory motifs (ITIMs) in their cytosolic tails, which suggests that they are able to perform inhibitory functions when they bind sialylated carbohydrates. In fact, many Siglecs are inhibitory receptors that mediate a variety of different inhibitory functions in the immune system, such as regulation of the inflammation mediated by damage-associated and pathogen-associated molecular patterns (DAMPs and PAMPs), promoting the tolerance of B lymphocytes and modulating the activation of dendritic cells and the activation of T cells [70, 74].

The receptor Siglec-E target of the *T. cruzi* mucins is a restricted leukocyte antigen mainly expressed on mouse phagocytic cells and on antigen-presenting cells (APCs) including macrophages and dendritic cells [75, 76]. Binding of *T. cruzi* to Siglec-E-expressing cells is followed by rapid mobilization of Siglec-E into the contact zone between parasite and host cells. This triggering of Siglec-E modulates the activity of dendritic cells, leading to lower production of IL-12, which is important for Th1 responses [72, 73]. In addition, triggering of Siglec-E on dendritic cell surfaces with cross-linking antibodies reduces the capacity of T cells to be activated and proliferate [72]. This phenomenon may contribute to the parasite-induced modulation of host immunity by damping T cell protective responses.

The capacity of *T. cruzi* mucins to modulate the adaptive immune response does not seem to be restricted to *T. cruzi*. The inefficient host immune response to cancer antigens is at least in part due to the presence of carcinoma-associated mucins [77–81]. However, the mechanisms involved in these effects of mucin-like molecules on the immune system are not well understood. In this connection, we have recently shown that the *T. cruzi* mucin is able to inhibit CD4^+^ T cell proliferation by inducing T cell anergy. We showed that exposure of CD4^+^ T cells to parasite mucins significantly reduced IL-2 secretion in response to TCR activation [69]. Furthermore, our findings indicate that the state of anergy induced in the T cells by the parasite mucins is not reversed by exogenous IL-2, implying that the IL-2 pathway is irreversibly impaired upon activation of CD4^+^ T cells in the presence of *T. cruzi* mucins. These inhibitory effects of *T. cruzi* mucins also extend to the other aspects of CD4^+^ T cell differentiation pathways, as we have shown that the parasite mucin inhibits the production of cytokines during TCR stimulation, including those known to protect against parasite infections, such as IFN-*γ* and TNF-*α* [69].

We have also asked whether the sialylation of the *T. cruzi* mucin influences the strength of its inhibition of CD4^+^ T cells. We found that the removal of the sialic acid terminal residues by neuraminidase treatment partially abolished the inhibitory effects of the mucin on CD4^+^ T cell proliferative responses, indicating a possible role for the sialic acid-binding Ig-like lectin receptors expressed by T cells in the inhibitory effects of the parasite mucins [69]. In fact, we showed for the first time that triggering of CD33 on CD4^+^ T cells with anti-Siglec E antibodies significantly inhibited the proliferation of stimulated T cells. We therefore propose that the T cell surface mucin receptor Siglec-E is implicated in the inhibition of T cell proliferation [69] (Figure 1).

When dissecting the signaling pathway of the *T. cruzi*-mediated inhibition of T cell responses, we found that the parasite mucin was able to induce G1 cell cycle arrest associated with upregulation of the cyclin D inhibitor p27(kip1) and downmodulation of cyclin D3 on activated CD4^+^ T cells [69]. p27 is a phosphatase regulator that participates in the G1 cell cycle arrest checkpoint [82–84]. In contrast, when CD4^+^ T cells were polyclonally activated in the presence of desialylated *T. cruzi* mucin the signaling profile was reversed as demonstrated by the upregulation of cyclin D3 and downmodulation of p27(kip1), a profile similar to that described for control TCR-activated T cells. These data indicate that *T. cruzi* mucins exert antiproliferative effects on CD4^+^ T cells, inducing G1 phase arrest by increasing the amount of p27(kip1), an immune modulatory effect that is potentiated by the sialic acid terminal residues of the parasite mucins [69] (Figure 1).

## 4. Concluding Remarks

Several independent studies have provided evidence that *T. cruzi* mucins are involved in T cell responses by affecting the activation, differentiation, and expansion of T cells. Our results indicate that Tc Muc mediates the inhibitory effects on CD4^+^ T expansion and cytokine production by blocking cell cycle progression in the G1 phase. We propose that the sialyl motif of Tc Muc is able to interact with sialic acid-binding Ig-like lectins (Siglecs) on CD4^+^ T cells, which may allow the parasite to modulate the immune system. It is likely that Siglec-E is involved in this effect. It is noteworthy that our studies revealed that the mucin derived from *T. cruzi* parasites upregulates the expression of the mitogen inhibitor p27(kip1) associated with the G1-phase cell cycle arrest, and this phenomenon is potentiated by the sialyl terminal residues of *T. cruzi* mucins which are important for its inhibitory effects on T cells. These findings point to an important feature of pathogen virulence as we show that *T. cruzi* modifies the cell cycle phase of T cells to make them anergic to antigenic stimulation during the acquisition of protective immunity. Actually other diseases such as virus infections and cancers modulate the cell cycle to serve their own purposes [85, 86]. For instance, expression of cell cycle proteins like cyclins or cyclin-dependent kinases can be modulated in order to interrupt the cell cycle at a phase that is advantageous for a virus to replicate [86]. Understanding these intricate effects on cell cycle checkpoints, which play a role in damping natural and acquire immunity to invading microorganisms, will help us to elucidate the interplay between the virulence factors of infectious pathogens and the host immune response to infection in illnesses such as Chagas disease.
